# Mental Fatigue and Executive Dysfunction in Patients with Cushing's Syndrome in Remission

**DOI:** 10.1155/2015/173653

**Published:** 2015-06-28

**Authors:** Eleni Papakokkinou, Birgitta Johansson, Peter Berglund, Oskar Ragnarsson

**Affiliations:** ^1^Institute of Medicine, Sahlgrenska Academy, University of Gothenburg, Gothenburg, Sweden; ^2^Department of Endocrinology, Sahlgrenska University Hospital, Blå stråket 5, 413 45 Gothenburg, Sweden; ^3^Institute of Neuroscience and Physiology, Sahlgrenska Academy, University of Gothenburg, Gothenburg, Sweden

## Abstract

Patients with Cushing's syndrome (CS) in remission often suffer from impaired quality of life and cognitive dysfunction. The primary aim was to investigate the occurrence of mental fatigue, characterized by mental exhaustion and long recovery time following mentally strenuous tasks, in patients with CS in remission. The secondary aim was to examine whether the newly developed parts C and D of the trail making test (TMT) are more sensitive, compared to the conventional parts A and B, to evaluate attention and executive function. This was a cross-sectional study including 51 patients with CS in remission and 51 controls. All subjects completed the self-administrated mental fatigue scale (MFS) and performed all four parts of the TMT. The patients had worse outcome on all components of the MFS except for sensitivity to noise. After adjustment for mental fatigue, depression, and anxiety, the patients performed worse only on part D of the TMT (*P* < 0.05). Mental fatigue is common in patients with CS in remission and can be captured by using the MFS. The most demanding part of the TMT, part D, is more useful to capture cognitive deficits in patients with CS in remission compared to the conventional parts A and B.

## 1. Introduction

Cushing's syndrome (CS) is caused by a prolonged and excessive exposure to glucocorticoids. The etiology of CS is heterogeneous, where the most common cause is the use of exogenous glucocorticoids. The most common cause of endogenous CS is adrenocorticotropic hormone (ACTH) producing pituitary adenoma (Cushing's disease (CD)). Other causes are cortisol-producing adrenal adenomas and ectopic ACTH producing tumors. The incidence of endogenous CS ranges from 0.7 to 2.4 per million population per year [1]. The chronic glucocorticoid exposure in CS affects almost all tissues in the human body and results in a wide range of symptoms like hypertension, central obesity, gonadal dysfunction, hirsutism, muscle weakness, and osteoporosis.

Mood disorders and cognitive impairment occur in 50–80% of patients with active CD [2]. Despite remission, as defined by biochemical control of the disease after treatment, a large number of patients suffer from impaired quality of life, fatigue, and cognitive dysfunction [3–7]. Also, van Aken et al. have shown that 45% of patients with CD in remission are depressed [8]. Recent studies have shown that the impaired cognitive function in patients with CS in remission not only occurs in the domains of short-term memory and executive function but also affects speed processing, working memory, verbal fluency, and reading speed [6, 7].

Mental fatigue is characterized by a mental exhaustion which appears especially during sensory stimulation or following mentally strenuous tasks [9]. Other typical features are the long recovery time that is needed for restoration of mental energy, irritability, impaired memory, and concentration as well as stress, sound, and light hypersensitivity. Mental fatigue is commonly observed after a traumatic brain injury, stroke, and infections or inflammatory diseases in the central nervous system [10]. Recently, the mental fatigue scale (MFS) has been developed for subjective evaluation of mental fatigue and related symptoms [9–11].

Our main hypothesis was that patients with CS in remission frequently suffer from mental fatigue that can be captured by using the MFS. The primary aim of this study was therefore to investigate the occurrence of mental fatigue by using the MFS in patients with CS in remission. The secondary aim was to study executive function and attention, using the trail making test (TMT) [12] and also to use the extended version (parts C and D) with an increased demand on divided attention. In our previous study, no difference was observed between the patients with CS in remission and controls when applying the conventional parts A and B of the test [7]. We hypothesized that the more demanding parts C and D of the TMT are more sensitive for evaluation of these domains of cognitive function.

## 2. Patients and Methods

### 2.1. Study Design

This was a cross-sectional, case-control study, designed to evaluate long-term outcome in patients previously treated for CS. The subjects were studied on three occasions, where medical history was reviewed, physical examination was performed, hormonal and psychiatric status was evaluated, and neuropsychological tests were performed as previously described [7]. In this part of the study, the results from the MFS and all parts of the TMT are presented.

### 2.2. Patients

The study group for the current analysis included 51 patients and 51 controls that completed the MFS and performed all parts of the TMT (A, B, C, and D). Validation of the diagnosis of CD and cortisol-producing adrenal adenomas was achieved by thorough review of the medical, chemical, radiological, and histopathological data from the time of diagnosis.

Thirty-nine patients (77%) had CD (35 women and 4 men). The primary treatment for CD was transsphenoidal pituitary surgery in 25 patients, pituitary radiotherapy in 6 patients, and bilateral adrenalectomy in 8 patients. Nine patients who were initially treated with transsphenoidal surgery needed additional treatment. Three received additional pituitary radiotherapy, five underwent a second transsphenoidal surgery, and one was treated by bilateral adrenalectomy. Of the patients who were initially treated by bilateral adrenalectomy, two had additional pituitary radiotherapy and two were treated with transsphenoidal surgery due to Nelson's tumor. Four of six patients who were treated with radiotherapy had subsequent radiotherapy and one transsphenoidal pituitary surgery.

All of the patients with cortisol-producing adrenal adenomas (*n* = 12) were women and were treated by unilateral adrenalectomy.

### 2.3. Controls

Controls were selected randomly from a population sample obtained from the Swedish tax agency. Potential controls matched for age and gender were contacted by an invitation letter. Those who responded were interviewed via telephone and subjects that matched the patient's education level and had no endocrine disease or chronic diseases known to affect cognitive function were included.

### 2.4. Evaluation of Hormone Status

Remission of CS was evaluated clinically and biochemically. The latter was achieved by measurement of 24 hr urinary free cortisol and by 1 mg overnight dexamethasone suppression test. The dexamethasone suppression test was performed in all patients except those operated with bilateral adrenalectomy. Serum cortisol concentration not greater than 50 nmol/liter after dexamethasone administration was considered an adequate suppression. Twenty-four-hour urinary free cortisol was collected in all patients and levels below the upper limit range were accepted. The thyroid, gonadal, and growth hormone (GH) status was evaluated clinically and by measurements of serum levels of free T_4_, TSH, testosterone, estrogen, dehydroepiandrosterone (DHEA) sulfate, androstenedione, gonadotropins, and IGF-1.

### 2.5. Assessment of Mental Fatigue

The self-administrated Swedish version of the MFS was used to evaluate mental fatigue. The scale has been used previously for evaluation of mental fatigue in patients with traumatic brain injuries and stroke [10]. The scale consists of 15 questions covering the most common symptoms of mental fatigue, that is, fatigue in general, lack of initiative, mental fatigue, memory problems, slowness of thinking, sensitivity to stress, increased tendency to become emotional, irritability, sensitivity to light and noise, decreased or increased sleep, and diurnal variations. Each item comprises examples of common activities to be related to four response alternatives (MFS is free to use and can be downloaded at http://mf.gu.se/). A rating of 0 reflects normal function, 1 indicates a problem, 2 indicates a pronounced symptom, and 3 indicates a maximal symptom. It is also possible to use graduations, that is, 0.5, 1.5, and 2.5 [9].

### 2.6. Psychiatric Evaluation

The Comprehensive Psychopathological Rating Scale (CPRS-A) was used to evaluate anxiety and depression [13].

### 2.7. Neuropsychological Testing: Trail Making Test A, B, C, and D

The TMT was designed to evaluate the speed of processing, sequence alternation, cognitive flexibility, visual search, motor performance, and executive function. During part A of the test, the subjects are asked to draw lines between 25 encircled numbers randomly written on a sheet of paper, consecutively connected in an ascending order (i.e., 1-2-3-4, etc.). In part B, the subjects have to alternate from numbers to letters while connecting them (i.e., 1-A-2-B-3-C, etc.) [12]. The tests should be performed as quickly and accurately as possible.

Parts C and D of the TMT were developed in order to assess higher demands during the testing [10]. These parts are constructed with three and four factors, respectively. Months were added in part C (i.e., 1-A-January-2-B-February, etc.), and both months and days of the week in chronological order in part D. In the latter, the order of letters and digits was switched (i.e., A-1-January-Monday-B-2-February-Thuesday, etc.) [10].

### 2.8. Ethical Considerations

Informed written consent was obtained from all the patients and controls. The local ethical committee of the University of Gothenburg, Sweden, approved the study. The study was conducted according to the Declaration of Helsinki.

### 2.9. Statistical Methods

Statistical analyses were performed with SPSS version 22. Data are presented as mean ± SD or median (interquartile range). We tested univariate hypotheses by using either parametric (Student's *t*-test) or nonparametric (Mann-Whitney *U* test) tests for continuous variables as appropriate to their distributions. Simple correlation was calculated by using Pearson's method. For proportions, Chi-square or Fisher's exact test was used as appropriate.

We used multiple linear regression with backward elimination to examine differences between patients and controls on the TMT tests while accounting for possible influences of mental fatigue (total score on the MFS) and affective disorder (CPRS-A subscales score for depression and anxiety). Multiple linear regression with backward elimination was used in order to evaluate differences between patients who received pituitary radiotherapy and patients who did not, taking into account age as a possible confounder. All variables that were not normally distributed were log-transformed before they were analyzed in the regression analyses. A two-tailed *P* value of < 0.05 was considered statistically significant.

## 3. Results

### 3.1. Subjects Characteristics

The mean age of patients and controls was 52.5 ± 14.6 and 53.6 ± 13.9 yrs. (*P* = 0.7), respectively. No differences were found between the groups concerning smoking habits or BMI (Table 1). There were more full-time employees in the control group compared to patients (*P* < 0.05).

All patients were in remission, confirmed by normal urinary free cortisol concentration and/or adequate suppression of serum cortisol on an overnight dexamethasone suppression test. The duration in remission was 12 (4–18) yrs. Twenty-one patients (41%) were on glucocorticoid replacement therapy and were receiving treatment with hydrocortisone (mean daily dose: 24 ± 11 mg). Twenty-one of 22 patients with GH deficiency were on GH replacement therapy. Three of four male patients had hypogonadotropic hypogonadism and received replacement therapy with testosterone. Three of fourteen premenopausal women had hypogonadotropic hypogonadism and received therapy with estrogen and progesterone. Fifteen patients were treated for central hypothyroidism and five for primary hypothyroidism.

### 3.2. Mental Fatigue Scale (MFS)

**Figure 1:**
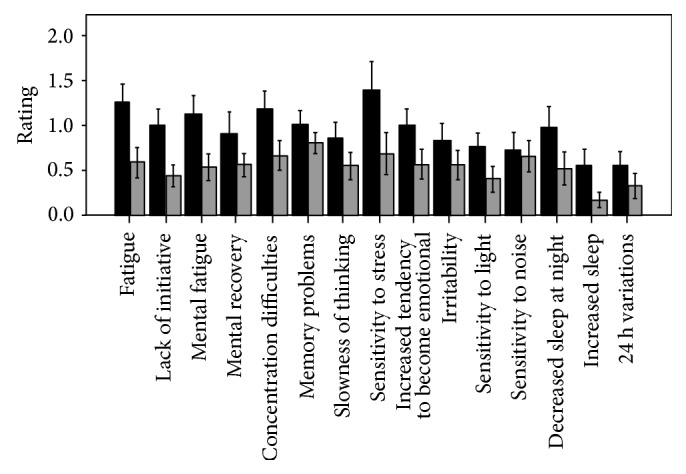
Bar charts showing the mean scores on the self-administrated mental fatigue scale in 51 patients with CS in remission (dark grey bars) and 51 controls matched for gender, age, and educational level (light grey bars). Significant difference was found between patients and controls in all items (*P* < 0.05) except for sensitivity to noise (*P* = 0.7). Error bars represent 95% confidence interval.

The mean total score on the MFS was 13.5 ± 7.4 in the patients compared to 7.8 ± 4.9 (*P* < 0.001) in controls. The patients had higher scores on all components of the MFS except for sensitivity to noise (Figure 1).

There was no difference in the mean total score on the MFS between patients treated for CD (mean: 13.3 ± 7.8) and cortisol-producing adrenal adenoma (mean: 16.1 ± 5.5; *P* = 0.3). Comparing patients with CD who had received radiotherapy and those who had not did not reveal difference in the mean total score on the MFS (11.5 ± 5.8 versus 14.3 ± 8.7; *P* = 0.3). Patients on glucocorticoid replacement therapy due to adrenal insufficiency did not perform worse compared to the patients with normal cortisol production (mean total score: 12.1 ± 7.9 versus 14.3 ± 7.7; *P* = 0.4). Also, patients with any hormone deficiency had similar total MFS total score (14.2 ± 7.6) compared to hormonally sufficient patients (13.3 ± 7.0; *P* = 0.7).

### 3.3. Trail Making Test A, B, C, and D

**Figure 2:**
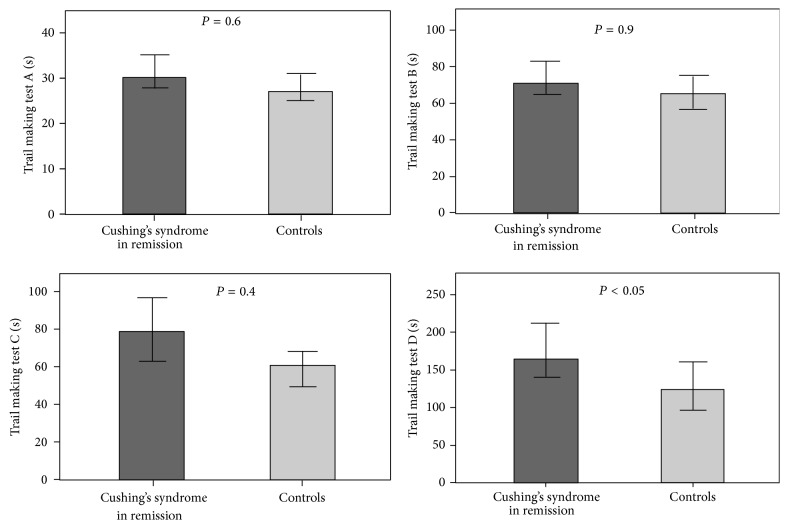
Bar charts showing the median time in seconds (95% confidence interval) on TMT parts A, B, C, and D in 51 patients with CS in remission (dark grey bars) and 51 controls matched for gender, age, and educational level (light grey bars). The *P* values are obtained by multiple linear regression analysis after adjustment for fatigue and scores for depression and anxiety.

The patients needed longer time to complete part A [30 (26–40) versus 27 (23–36) seconds; *P* < 0.05], part C [80 (56–119) versus 62 (46–96) seconds; *P* < 0.05], and part D [165 (122–236) versus 124 (84–191) seconds; *P* < 0.05] of the TMT (Figure 2). No difference was seen in part B [71 (58–98) versus 65 (50–96) seconds; *P* = 0.16] between the groups.

There was a positive correlation (patients and controls analyzed together) between total score on the MFS and TMT part A (*r* = 0.34, *P* = 0.001), part B (*r* = 0.20, *P* < 0.05), part C (*r* = 0.22, *P* < 0.05), and part D (*r* = 0.22, *P* < 0.05). There was also a positive correlation between score for depression and TMT part A (*r* = 0.20, *P* < 0.05) and between score for anxiety and part A (*r* = 0.34, *P* = 0.001) and part C (*r* = 0.22, *P* = 0.05). After adjustment for mental fatigue, depression, and anxiety, the patients performed worse on part D of the TMT (*P* < 0.05) but not on parts A, B, and C.

No difference was found in median total score on any parts of the TMT regarding etiology of the CS (part A, *P* = 0.7; part B, *P* = 0.8; part C, *P* = 0.4; part D, *P* = 0.3) or glucocorticoid replacement therapy (part A, *P* = 0.8; part B, *P* = 0.9; part C, *P* = 0.3; part D, *P* = 0.4). After adjustment for age, patients who had received pituitary radiotherapy performed worse on part B [87 (79–115) seconds] compared to those who had not [63 (53–75) seconds; *P* < 0.01].

## 4. Discussion

The results in this study demonstrate that mental fatigue is common in patients with CS in remission and that the self-administrated questionnaire MFS is a useful tool for evaluation of this clinically relevant condition.

The MFS was originally developed in order to evaluate mental fatigue and thereof related symptoms that patients with various brain disorders often experience. The MFS takes into account a number of variables that affect mental fatigue such as sleep and emotional and cognitive symptoms and can also be used to distinguish between mental and general fatigue [9]. In previous studies, quality of life (QoL) has been evaluated by various general questionnaires as well as the disease specific questionnaire CushingQoL [14, 15]. Although the CushingQoL is a valuable questionnaire for patients with active disease and for prospective evaluation of changes following treatment, its value in cross-sectional studies on patients in remission can be questioned. The MFS is possibly better than general QoL questionnaires since it captures symptoms that are of great importance for general health in patients treated for CS and provides at least additional and valuable information on the patients' well-being in comparison to the disease specific questionnaire [15]. In addition, one of the aims in the longitudinal follow-up of patients treated for CS is to improve QoL [4]. The MFS is probably a valuable instrument for this purpose and can be used to detect mental fatigue at diagnosis and during the follow-up to objectively compare changes before and after treatment. A cut-off score of 10.5 is suggested for MFS [11].

Another interesting finding in this study is that the most demanding part of the TMT, part D, seems to be more useful to capture cognitive deficits in patients with CS in remission compared to the conventional parts A and B. Parts A and B of the TMT are well established and evaluate speed performance, visual scanning, and divided attention. TMT parts C and D were developed to evaluate higher cognitive demands, such as dual tasks [10]. Interestingly, there is a positive correlation between total score for MFS and TMT, which indicates that the subjects with higher fatigability need longer time to complete TMT. In the current study, after adjustment for mental fatigue, depression, and anxiety, the patients performed worse only on part D of the test. This indicates that part D or other complex cognitive tests can be more sensitive to reveal difficulties in executive functioning and cognitive flexibility in patients with CS to capture subtle cognitive deficits.

The reason for the irreversibility of mental fatigue and cognitive impairment after treatment of patients with CS is to date still unclear. Compared to patients treated for nonfunctioning pituitary adenomas, patients with CD in remission perform worse in certain aspects of executive functioning and memory and report a higher prevalence of psychopathology [6, 16]. This indicates that the effect of previous hypercortisolism on cognitive function and generally on the central nervous system is irreversible. Also, the pattern of neurodegenerative and neuroinflammatory biomarkers in cerebrospinal fluid between patients with CS in remission and healthy controls is not different, indicating that the pathophysiology of cognitive dysfunction in CS is not the same as in other common neurodegenerative disorders [17]. So far, there are no known predictive factors for the development of cognitive impairment in these patients. We have, however, found that polymorphisms in the 11*β* HSD type 1 gene, coding for an enzyme involved in GC metabolism, and polymorphism in the glucocorticoid receptor gene (*NR3C1*) were associated with impaired cognitive dysfunction and fatigue in patients with CS [18]. Also, in one study, it was found that persistent hypocortisolism necessitating replacement therapy was associated with worse cognitive function [19]. This association could not be confirmed in our study.

The main strengths of this study are the well characterized study population and the implementation of novel tests for evaluation of symptoms that are highly relevant for patients with CS syndrome in remission. Another strength is that we adjusted for several factors in the statistical analysis of the TMT, that is, fatigue, anxiety, and depression, and thereby minimized the influence of potential confounders. A limitation is that the patients are a heterogeneous group in terms of etiology, hormone deficiencies, previous treatments, age at diagnosis, and duration in remission. It has been suggested that age, age at diagnosis, gender, and hypopituitarism can be independent determinants of QoL after successful treatment of Cushing's disease [8]. However, reported results are not consistent [7]. We found that patients with CS in remission experience mental fatigue and have impaired executive function, which is not associated with etiology of CS, radiotherapy, or glucocorticoid replacement therapy.

## 5. Conclusions

In conclusion, mental fatigue is common in patients with CS in remission. These results indicate that we should consider including MFS and more demanding cognitive tests in order to evaluate mental fatigue, attention, and executive functioning in patients with CS. It is important to determine even subtle cognitive impairment which can lead to reduced quality of life, depression, and invalidating.

## Figures and Tables

**Table 1:** Background characteristics and sociodemographic status in 51 patients with CS in remission and 51 healthy controls matched for age, gender, and educational level.

	Patients	Controls	*P*
Age at follow-up (yr)	52.5 ± 14.6	53.6 ± 13.9	0.7
Age at diagnosis (yr)	36.4 ± 13.6		
Duration of remission (yr)	12 (4–18)		
BMI	27.0 ± 6.6	25.9 ± 5.2	0.4
Education level (%)			0.9
Elementary school	22	22	
Upper secondary education	51	55	
University education	27	23	
Smoking habits (%)			0.9
Nonsmoker	58	53	
Ex-smoker	32	37	
Smoker	10	10	
Marital status (%)			0.9
Single/divorced	27	27	
Married	69	71	
Widow	4	2	
Number of children	2 (0–2)	2 (1-2)	0.3
Employment (%)			0.09
Full-time	34	51	
Part-time	30	20	
Sick leave/disability pension	10	0	
Retirement	26	29	

## References

[1] Newell-Price J., Bertagna X., Grossman A. B., Nieman L. K. (2006). Cushing's syndrome. *The Lancet*.

[2] Dorn L. D., Burgess E. S., Friedman T. C., Dubbert B., Gold P. W., Chrousos G. P. (1997). The longitudinal course of psychopathology in Cushing's syndrome after correction of hypercortisolism. *Journal of Clinical Endocrinology and Metabolism*.

[3] Resmini E., Santos A., Gómez-Anson B. (2012). Verbal and visual memory performance and hippocampal volumes, measured by 3-Tesla magnetic resonance imaging, in patients with Cushing's syndrome. *The Journal of Clinical Endocrinology and Metabolism*.

[4] Ragnarsson O., Johannsson G. (2013). Cushing's syndrome: a structured short- and long-term management plan for patients in remission. *European Journal of Endocrinology*.

[5] Forget H., Lacroix A., Cohen H. (2002). Persistent cognitive impairment following surgical treatment of Cushing's syndrome. *Psychoneuroendocrinology*.

[6] Tiemensma J., Kokshoorn N. E., Biermasz N. R. (2010). Subtle cognitive impairments in patients with long-term cure of Cushing's disease. *The Journal of Clinical Endocrinology & Metabolism*.

[7] Ragnarsson O., Berglund P., Eder D. N., Johannsson G. (2012). Long-term cognitive impairments and attentional deficits in patients with Cushing's disease and cortisol-producing adrenal adenoma in remission. *The Journal of Clinical Endocrinology and Metabolism*.

[8] van Aken M. O., Pereira A. M., Biermasz N. R. (2005). Quality of life in patients after long-term biochemical cure of Cushing's disease. *The Journal of Clinical Endocrinology and Metabolism*.

[9] Johansson B., Starmark A., Berglund P., Rödholm M., Rönnbäck L. (2010). A self-assessment questionnaire for mental fatigue and related symptoms after neurological disorders and injuries. *Brain Injury*.

[10] Johansson B., Berglund P., Rnnbck L. (2009). Mental fatigue and impaired information processing after mild and moderate traumatic brain injury. *Brain Injury*.

[11] Johansson B., Rönnbäck L. (2013). Evaluation of the mental fatigue scale and its relation to cognitive and emotional functioning after traumatic brain injury or stroke. *International Journal of Physical Medicine and Rehabilitation*.

[12] Reitan R. M., Wolfson D. (1985). *The Halstead-Reitan Neuropsychological Test Battery. Theory and Clinical Interpretation*.

[13] Asberg M., Montgomery S. A., Perris C., Schalling D., Sedvall G. (1978). A comprehensive psychopathological rating scale. *Acta Psychiatrica Scandinavica, Supplement*.

[14] Lindsay J. R., Nansel T., Baid S., Gumowski J., Nieman L. K. (2006). Long-term impaired quality of life in Cushing's syndrome despite initial improvement after surgical remission. *The Journal of Clinical Endocrinology and Metabolism*.

[15] Webb S. M., Badia X., Baarahona M. J. (2008). Evaluation of health-related quality of life in patients with Cushing's syndrome with a new questionnaire. *European Journal of Endocrinology*.

[16] Tiemensma J., Biermasz N. R., Middelkoop H. A. M., van der Mast R. C., Romijn J. A., Pereira A. M. (2010). Increased prevalence of psychopathology and maladaptive personality traits after long-term cure of Cushing's disease. *The Journal of Clinical Endocrinology and Metabolism*.

[17] Ragnarsson O., Berglund P., Eder D. N. (2013). Neurodegenerative and inflammatory biomarkers in cerebrospinal fluid in patients with Cushing's syndrome in remission. *European Journal of Endocrinology*.

[18] Ragnarsson O., Glad C. A., Berglund P., Bergthorsdottir R., Eder D. N., Johannsson G. (2014). Common genetic variants in the glucocorticoid receptor and the 11*β*-hydroxysteroid dehydrogenase type 1 genes influence long-term cognitive impairments in patients with Cushing's syndrome in remission. *The Journal of Clinical Endocrinology & Metabolism*.

[19] Psaras T., Milian M., Hattermann V., Will B. E., Tatagiba M., Honegger J. (2011). Predictive factors for neurocognitive function and quality of life after surgical treatment for Cushing's disease and acromegaly. *Journal of Endocrinological Investigation*.

